# The trends in land surface heat fluxes over global monsoon domains and their responses to monsoon and precipitation

**DOI:** 10.1038/s41598-020-62467-0

**Published:** 2020-04-01

**Authors:** Jian Zeng, Qiang Zhang

**Affiliations:** 1 College of Atmospheric Sciences, Lanzhou University, Lanzhou, China; 2 Key Laboratory of Arid Climatic Change and Disaster Reduction in Gansu Province, Key Open Laboratory of Arid Climatic Change and Disaster Reduction in CMA, Institute of Arid Meteorology, CMA, Lanzhou, China; 3 Gansu Provincial Meteorological Bureau, Lanzhou, China

**Keywords:** Atmospheric dynamics, Attribution

## Abstract

The climatology, trends and leading modes of land surface latent heat flux (LHF) and sensible heat flux (SHF) as well as their responses to monsoon and precipitation in global land monsoon domains are presented. During the past three decades, LHF and SHF have generally undergone a rising and decreasing trend (that is, (LHF+, SHF−)), respectively, in Asian, North African, Austrian, and South American monsoon domains. Moreover, the increasing rate of LHF was higher than the decreasing rate of SHF, which causes a decreased trend in Bowen ratio. Two other dominant trend patterns, (LHF−, SHF−) and (LHF+, SHF+), are observed in South African and South American monsoon domains, respectively. The trends in LHF and SHF are closely linked to increasing global monsoon intensity and precipitation, especially for the monsoon domain that has annual precipitation lower than 1300 mm yr^−1^. Singular value decomposition (SVD) analyses show that monsoon strength explains 25.2% and 22.2% total covariance of LHF and SHF respectively in the first modes, and that precipitation slightly raises the percentages up to 27.8% and 24% respectively. The increasing monsoon and precipitation on one hand favor more land surface available energy being converted into LHF; on the other hand they enhance the LHF by increasing the land surface net radiation. Moreover, remarkable phase shifts in LHF and SHF are observed for monsoon domains during late-1990s, which are in phase with those of precipitation and monsoon strength. The intensifying LHF and precipitation indicate the acceleration of hydrological cycle in global terrestrial monsoon domains.

## Introduction

Land surface heat fluxes, including latent heat flux (LHF) and sensible heat flux (SHF), link the land surface with the atmosphere by transporting land surface energy and moisture into the atmosphere. Therefore, they are critical parts of water and energy cycles, and govern the land-atmosphere interactions.

Land surface conditions including soil moisture and green vegetation fractions significantly impact the land-atmosphere linkage by dominating the partitioning of land surface available energy into LHF and SHF. The partitioning of land surface available energy is often described by the ratio of SHF to LHF, known as the Bowen ratio. Bowen ratio alone can reflect the relative magnitudes of SHF and LHF as well as the land surface thermal-hydrologic properties. Wetter land surface and larger vegetation fractions favor higher LHF and smaller SHF, resulting in a smaller Bowen ratio. For instance, the Bowen ratio is found to be 0.61 during dry winter season but it reduces to 0.18 during summer monsoon season for cropland^1^. For irrigation-dependent agricultural region, the ratio can even reach as low as 0.01^2^. The energy partitioning that contains the information of land surface thermal-hydrologic properties further exerts influences on atmosphere by initially modifying boundary layer structure including the stability and height, which finally significantly affects atmospheric circulations, precipitation and climate on local, regional or larger scales^3–6^. Therefore, the land surface energy partitioning is closely related to climate variability.

Over land, however, the thermal-hydrologic properties of land surface are significantly regulated by precipitation. LHF and SHF are thus closely linked to precipitation. Moreover, precipitation and the moisture transported via LHF (or evapotranspiration) are the two key components of land surface hydrological balance. The LHF-precipitation feedback is the most uncertain part of soil moisture–precipitation feedback, as well as the most difficult to ascertain given the number of processes involved^7^. Therefore, the LHF and precipitation couplings are the crucial step in the soil moisture–precipitation feedback loop, and require further investigations.

The global monsoon domains, located primarily in tropics and characterized by abundant precipitation, are crucial sources of land surface heat fluxes. Global monsoon is a powerful system and exerts prominent influences on regional and global weather and climate. Over half of the globe’s population, most in developing countries, lives under the impacts of monsoon-dominated climates^8^. In these regions, monsoonal precipitation provides the majority of fresh water for agriculture and ecosystems. With the global warming, the global monsoonal precipitation has significantly increased in the recent decades^9–11^. In future, the annual mean and range of global monsoonal precipitation and the percentage of summer rainfall are projected to continue to intensify at a significant level over most of the global regions^12^. The increasing precipitation should alter the land surface energy partitioning and thus, the water and energy cycles and the linkage between the land and the atmosphere, although to what extent remains unknown. Furthermore, land surface energy fluxes, in turn, are crucial for understanding the increasing trends in monsoonal precipitation.

However, the variability and long-term trends of land surface heat fluxes are not yet well investigated in global monsoon domains^13^. The responses of land surface heat fluxes to monsoon and monsoonal precipitation is not clear either. This is primarily due to a lack of reliable observations, although great efforts have been made to estimate the LHF and SHF. Various satellite remote sensing approaches have been applied to characterize their spatio-temporal variations, but so far only short-duration products are available and have the problems of questionable accuracy^14^. In the past two decades, eddy covariance (EC) measurement networks have been established and land surface heat fluxes from these observations networks are more reliable^15,16^. But they are limited by sparse spatial resolution. Jung *et al*. have recently upscaled the current FLUXNET observations of LHF and SHF using machine learning technique and model tree ensembles^17^. Cross-validation analyses show that the gridded FLUXNET have good performance in representing among-site flux variability and seasonal patterns^18^. Therefore, this dataset offers an opportunity to study the variability of the terrestrial LHF and SHF, and helps understand the water and energy cycling in global monsoon domains.

This paper primarily aims to survey the spatial patterns of land surface energy partitioning over global monsoon domains, investigate their variations and trends under increasing precipitation and global warming during the past three decades, and discuss the response of land surface energy partitioning to monsoon and precipitation.

## Method and Data

### Determination of global monsoon domains over land

**Figure 1:**
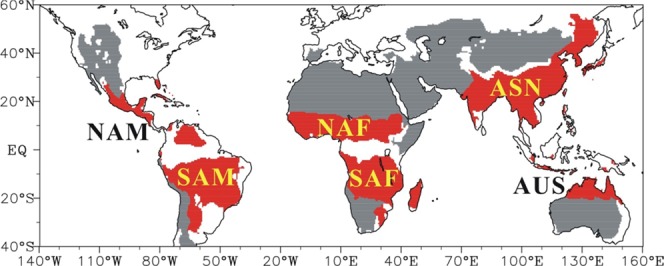
The global monsoon domains on land (red). The drylands are shaded in grey. Drylands are defined as regions where the annual precipitation P < 600 mm and ratio of annual precipitation to potential evapotranspiration P/PET < 0.65^45^.

Conventionally, the terminology “monsoon” was defined by the seasonal shift of prevailing winds between winter and summer^19^. The term now also increasingly refers to regions where there is a clear precipitation contrast between rainy phase (summer) and dry phase (winter)^20^. Therefore, the global monsoon domains over land are defined by the annual precipitation range followingWang and Ding^21^. Specially, the global monsoon domains refer to the regions where the annual range of precipitation between wet and dry seasons exceeds 2.5 mm day^−1^ and the wet seasonal precipitation contributes more than 55% of the total annual precipitation. The wet season denotes May–September (November–March) for the Northern (Southern) Hemisphere, while the dry season is the opposite. The global monsoon domains defined by annual precipitation range are shown in Fig. 1. They can be divided into six sectors, including the Asian (ASN), North African (NAF), and North American (NAM) monsoon sectors in the Northern Hemisphere (NH), as well as the Austrian (AUS), South African (SAF), and South American (SAM) monsoon sectors in the Southern Hemisphere (SH).

### Data

**Figure 2:**
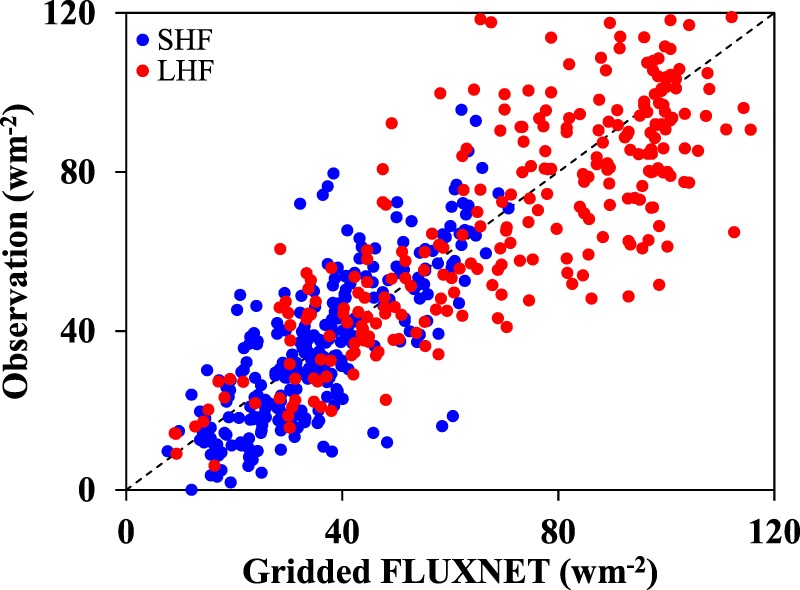
Comparisons of monthly LHF and SHF between the gridded FlUXNET and observations from nine *in-situ* sites.

The land surface latent heat flux (LHF) and sensible heat flux (SHF) are provided by the gridded FLUXNET dataset. The gridded FLUXNET dataset is derived from continuous *in-situ* measurements of FLUXNET, remote sensing and meteorological observations based on model tree ensembles^18^. This dataset covers a period of 30 years (1982–2011) at 0.5° × 0.5° spatial and monthly temporal resolution. Here the gridded FLUXNET dataset is validated by observations from eddy covariance system at nine land surface *in-situ* sites in monsoon domains (Table 1). These representative observation sites are well maintained and the observed LHF and SHF are under strict quality control. Necessary procedures for corrections and quality control of the turbulent fluxes are implemented during post-field data processing. Their detailed information is listed in Table 1. As shown in Fig. 2, the gridded FLUXNET dataset performs well in representing the LHF and SHF. The correlation coefficients between the observations and the gridded FLUXNET are 0.78 and 0.76 for LHF and SHF, respectively, which is in line with the previous studies^22,23^. The root mean square error (RMSE) is 1.06 and 1.70 MJ m^−2^ day^−1^ for LHF and SHF, respectively. The RMSE for LHF is higher than the 0.7 MJ m^−2^ day^−1^ of Yang *et al*. partly due to sample size and observation errors^22^, but it is acceptable for climatology and trend analyses. Moreover, cross-validation analyses also show that gridded FLUXNET SHF and LHF have good performance in representing among-site flux variability and seasonal patterns^18^. Therefore, the gridded FLUXNET dataset provides good and reliable estimates of LHF and SHF. Notably, the ground heat flux is not available from the gridded FLUXNET dataset. But the ground heat flux has opposite signs during daytime and nighttime, and during warm and cold seasons. Since the annual data is used, the impact of ground heat flux may be negligible.

**Table 1:** Details of the observation sites.

Site name	Latitude (°)	Longitude (°)	Vegetation type	Observation period
Kennedy Space Center Scrub Oak	28.609	−80.672	Closed shrubland	2000–2006
Kennedy Space Center Slash Pine Flatwoods	28.458	−80.671	Evergreen needleleaf forest	2002–2003
Mize	29.765	−82.245	Evergreen needleleaf forest	1998–2004
Freeman Ranch Mesquite Juniper	29.950	−97.996	Woody savanna	2004–2006
Donaldson	29.755	−82.163	Evergreen needleleaf forest	1999–2004
Dinghushan	23.150	112.500	Evergreen broadleaf forest	2003–2002
Qianyanzhou	26.730	115.500	Evergreen needleleaf forest	2003–2003
Xishuangbanna	21.900	102.267	Evergreen broadleaf forest	2003–2004
Yucheng	36.858	116.640	Cropland	2003–2005

Monthly precipitation is taken from the Climatic Research Unit Timeseries (CRU TS) compiled by the CRU^24^. In this paper, the version CRU TS 4.01 is used, which was released in September 2017. This dataset, based on analysis of over 4000 individual weather station records, spans the period January 1901 to December 2016 at 0.5° × 0.5° spatial resolution and on monthly scale. Compared to its previous versions 3.xx, this new version switches from triangulated linear interpolation to Angular Distance Weighting for gridding the monthly anomalies, which allows total control over how station observations are selected for gridding. Harris *et al*. compared the precipitation data from CRU to that from the Global Precipitation Climatology Centre (GPCC) dataset^24^. Close agreement for precipitation was demonstrated between CRU and the GPCC dataset in many sub-continental regions, except for the first 50 years (1901–1950). CRU dataset has been used for drought assessments^25–27^, climate change assessments^28,29^, and climate trend^30^.

### Simple measurement of global monsoon strength

**Figure 3:**
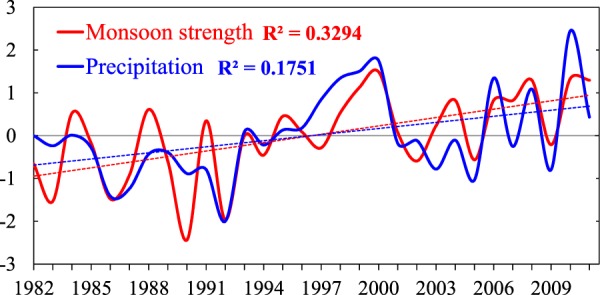
The time series of normalized monsoon strength (red solid line) and precipitation (blue solid line) in global land monsoon domains. The dashed lines are the linear trends for monsoon strength and precipitation, which are statistically significant at 99% and 95% confident level, respectively. The determination coefficients (*R*^2^) for the linear fitting are also presented.

Similar to Hsu *et al*.^9^, here we take the annual range of precipitation between summer/warm and winter/cold seasons as a measurement of monsoon strength. But we find this measurement may lead to mislead in the strength for monsoon sectors that have abundant rainfall for both summer and winter seasons. For example, the strongest monsoon is observed in sectors SAM and NAM (that is, largest annual range of precipitation) based on annual range of precipitation, which is inconsistent with the conventional notions (Fig. S1). Therefore, we improve the measurement approach by excluding the impact of background precipitation. The annual range is normalized by the climatological annual precipitation to define the monsoon strength δ (Eq. 1). Under the improved definition, the monsoon strength is 1.94, 1.65, 1.64, 1.45, 1.37, and 1.18 for AUS, NAF, SAF, ASN, SAM and NAM, respectively. The monsoon is weakest in SAM and NAM and strongest in AUS. The NAF, SAF and ASN are in-between. Since the annual range of precipitation (in mm d^−1^) is adopted to define monsoon domains, δ can to large extent reflect the intensity of monsoon. According to the definition, a large (small) δ means active (inactive) monsoon. Figure 3 presents its temporal variation along with precipitation in global land monsoon domains. Both the monsoon strength and precipitation show a statistically significant enhancement in the past 30 years, at 99% and 95% confident level, respectively.

1 $$\delta =\frac{{P}_{summer}-{P}_{winter}}{\bar{{P}_{climatology}}}$$

Here δ denotes the monsoon strength. *P*_*summer*_ and *P*_*winter*_ are the total precipitation for summer and winter, respectively. $\bar{{P}_{climatology}}$ is the long-term mean annual precipitation.

In fact, the annual range of precipitation has been accepted and frequently used to defined the monsoon strength in previous works^31,32^. The definition of the global monsoon strength based on precipitation essentially reflects the seasonal change of prevailing wind^32^. Moreover, global monsoon strength based on precipitation significantly (r = 0.85) correlates with monsoon intensity measured by the vertical shear of zonal wind and varies in phase with Hadley circulation intensity (Wang *et al*., 2013). Therefore, δ can provide reasonable and simple measurement of monsoon activity, and reflect both of the seasonal changes of prevailing wind and monsoonal precipitation.

## Climatology of LHF and SHF

**Figure 4:**
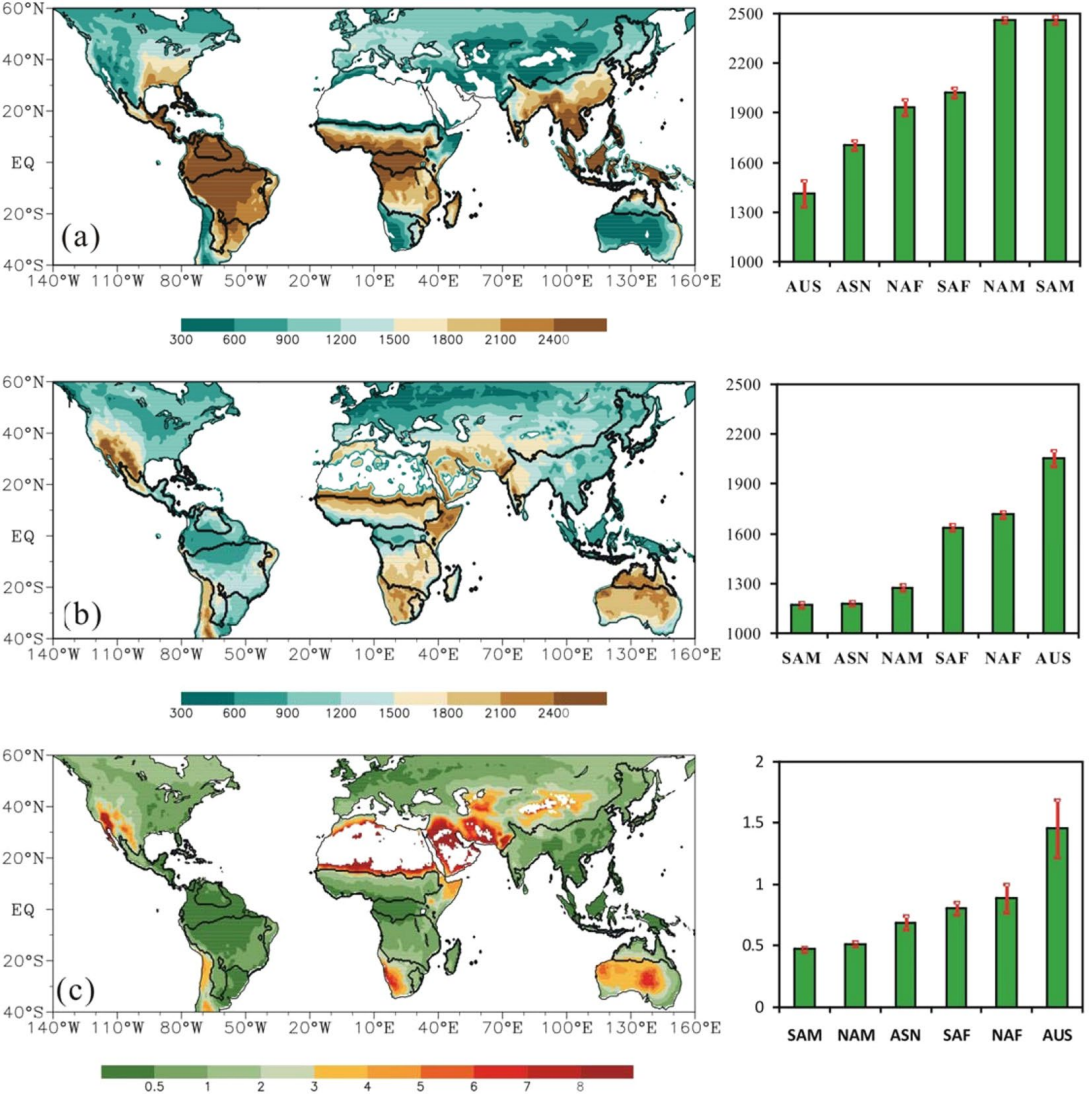
Climatology of (**a**) LHF, (**b**) SHF, and (**c**) Bowen ratio. The global monsoon domains are outlined by bold contours and the histograms show the regional averages of six monsoon sectors. The unit of LHF and SHF is MJ m^−2^ yr^−1^. The red bars in the columns are the standard deviations.

The global monsoon domains are crucial sources of land surface heat fluxes. Figure 4 shows the long-term annual mean LHF, SHF, and Bowen ratio (the ratio of SHF to LHF) during 1981 to 2011. As expected, the land surface available energy is largely consumed by the LHF in monsoon domains. The mean annual LHF for monsoon domain is 2000.5 MJ m^−2^ yr^−1^, comparing with 1506.6 MJ m^−2^ yr^−1^ of SHF. But significant regional differences in land surface energy partitioning are observed among six monsoon domains. The monsoon sectors SAM and NAM have the largest LHF, followed by SAF and NAF, ASN and AUS. The mean annual LHF in six monsoon sectors ranges from 1411.7 to 2462.7 MJ m^−2^ yr^−1^. In contrast, AUS has the highest SHF, followed by NAF and SAF, NAM, ASN and SAM. The mean annual SHF ranges from 1170.6 to 2055.8 MJ m^−2^ yr^−1^. Therefore, Bowen ratio is highest in AUS, followed by NAF and SAF, ASN, NAM and SAM. Notably the long-term mean SHF is larger than LHF in AUS and thus, AUS is the only monsoon sector that has Bowen ratio above 1, which seems contradictory to the fact that AUS has the strongest monsoon. In fact, a monsoon includes a dry phase and a rainy phase. The definitions of monsoon and monsoon strength emphasize the seasonal change but not its rainy season alone. This can also explain why NAM and SAM has the weakest monsoon but its Bowen ratio is lowest. It is evident that the differences in LHF, SHF and Bowen ratio among six sectors are continental. SAF and NAF, located in the African continent, have similar LHF, SHF and Bowen ratio, and the same case for SAM and NAM which belong to the American continent. But there exist significant differences between African and American monsoon sectors, which reflect the contribution of radiation budget and background climates on land surface heat exchanges. In fact when LHF, SHF and Bowen ratio are normalized by their maximums in each monsoon sector, the regional differences in normalized LHF, SHF, and Bowen ratio among six monsoon sectors are negligible.

**Figure 5:**
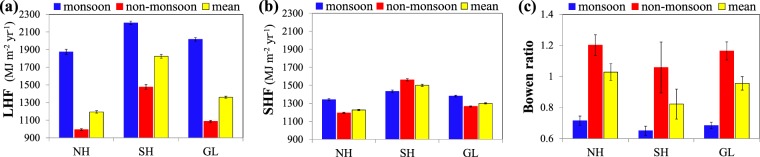
Long-term mean of annual (**a**) LHF, (**b**) SHF, and (**c**) Bowen ratio for monsoon, non-monsoon, and global domains during 1982–2011.

Besides, high LHF is one of the prominent features that separate monsoon from most of non-monsoon regions. As shown in Fig. 5, monsoon domains have much larger LHF than non-monsoon regions, while no significant contrast in SHF is seen. Therefore, Bowen ratio is much smaller in monsoon regions.

## Trends in LHF and SHF

**Figure 6:**
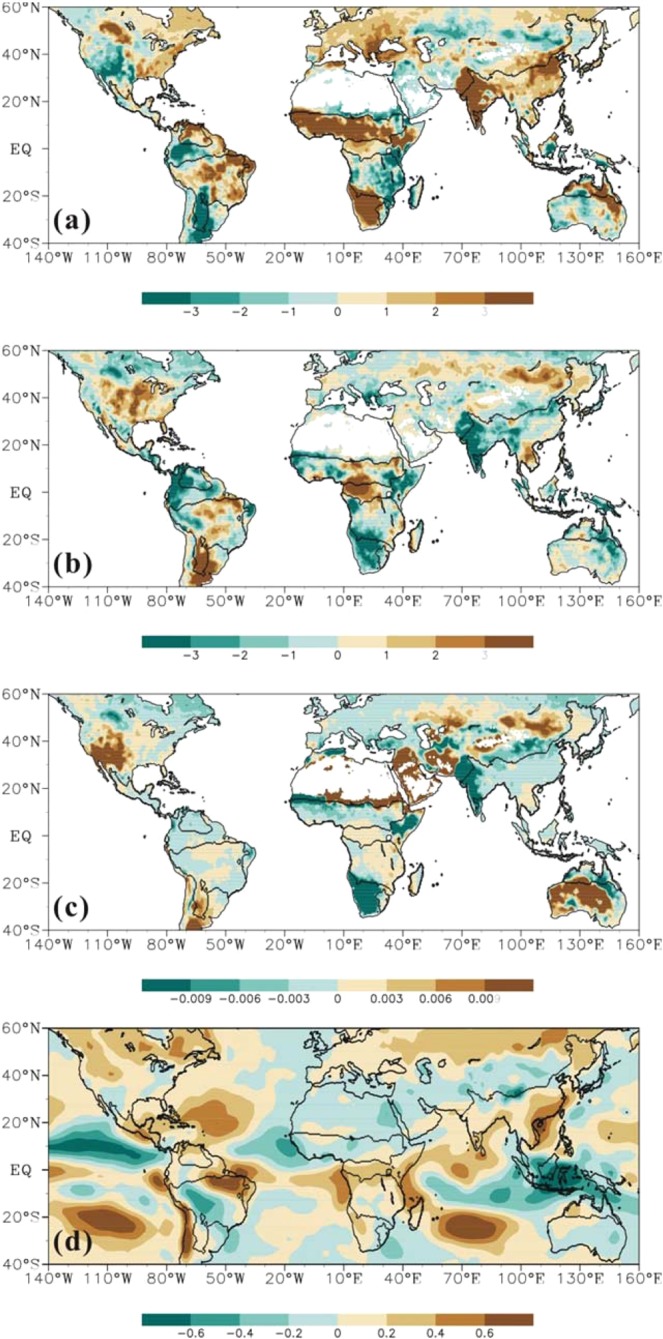
Trends for (**a**) LHF (in MJ m^−2^ yr^−1^ yr^−1^), (**b**) SHF (in MJ m^−2^ yr^−1^ yr^−1^), (**c**) Bowen ratio (in yr^−1^), and (**d**) net radiation (in J m^−2^ yr^−1^ yr^−1^) during 1982–2011. The net radiation dataset is derived from NCEP.

**Figure 7:**
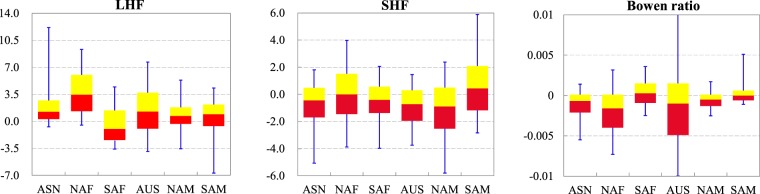
Box-and-whisker plots of trends for LHF (in MJ m^−2^ yr^−1^ yr^−1^), SHF (in MJ m^−2^ yr^−1^ yr^−1^), and Bowen ratio (in yr^−1^) within six monsoon domains during 1982–2011.

With the global warming, land surface fluxes show obvious trends in global monsoon domains. Figure 6 displays the spatial pattern of linear trends in LHF, SHF and Bowen ratio for the past 30 years (1982–2011). Increased trends in LHF are dominant in all monsoon sectors except SAF. More than 82% of land areas in ASN and NAF undergo an increase in LHF, and the fractions for AUS, NAM and SAM are 63%, 69% and 67% respectively. However, the percentage of land with rising trend is only 32% in SAF, indicating most areas of SAF had experienced a decrease in LHF. The median trends are 1.25, 3.47, −0.98, 1.26, 0.71, and 0.92 MJ m^−2^ yr^−1^ yr^−1^ for ASN, NAF, SAF, AUS, NAM, and SAM, respectively (Fig. 7). As for SHF, both the decreased and increased trends take up a significant portion in monsoon regions, but the decreased trend seems more dominant. About 61% area is in decreased trend of SHF for ASN, SAF, AUS, and NAM. More than 50% area in SAM and NAF undergo upward trends in SHF. The median trends in SHF are −0.45, −0.41, −0.72, −0.90, −0.01, and 0.44 MJ m^−2^ yr^−1^ yr^−1^ for ASN, SAF, AUS, NAM, NAF, and SAM, respectively. It is apparent that trends in SHF are in the opposite direction to trends in LHF for most of monsoon domains, which is expected because LHF will cool the land surface and narrow the land-atmosphere temperature difference. Moreover, the decreasing rates of SHF are much smaller than the increasing rate of LHF in monsoon domains, which implies the increased LHF is only partially from the decreased SHF and the remaining may be associated with an increase in land surface net radiation. As shown in Fig. 6d, the land surface net radiation derived from NCEP^33^ has undergone a rising trend in most of global monsoon domains over the past 30 years, which is in concert with previous study that the surface net radiation over land may have increased by about 2 W m^−2^ decade^−1^ during 1986 and 2000^34^. This may be also partially attributed to the global brightening since 1990s^35^. The Bowen ratio thus weakens for most of monsoon domains because of the faster increasing rate in LHF than the decreasing rate in SHF. The exceptions are the SAF and SAM. Bowen ratio shows an increased trend in about 58% area in SAF due to the weaker decreased rate of SHF than that of LHF. However in SAM, where the increased trends are dominant for both LHF and SHF, the percentages for upward and downward trends in Bowen ratio are almost equally shared. The median trends in Bowen ratios are −0.0007, −0.0016, 0.0003, −0.001, −0.0005, and 0.0 yr^−1^ for ASN, NAF, SAF, AUS, NAM and SAM, respectively.

**Figure 8:**
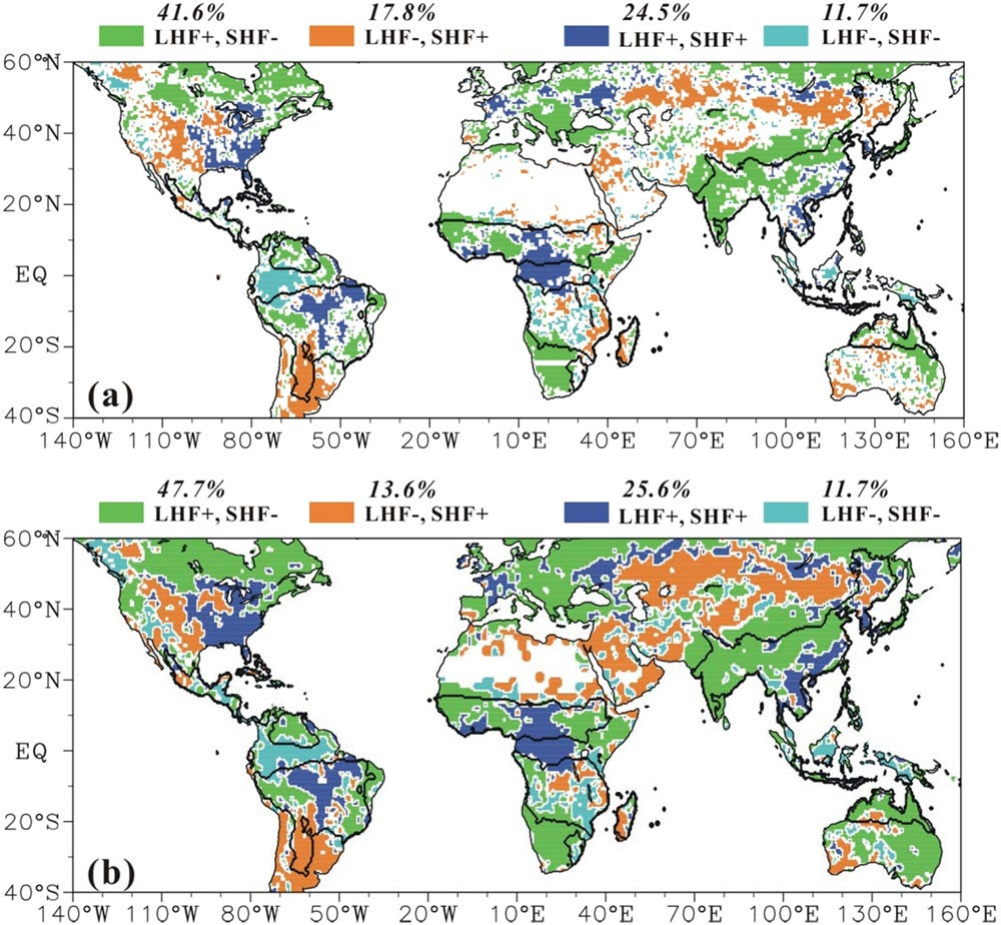
Statistics of the trend patterns in global terrestrial monsoon domains based on the (**a**) linear trends in LHF and SHF and (**b**) the primary SVD modes of LHF and SHF. The sign “+” denotes increasing trend, and “-” for decreasing trend.

Consequently, there are three dominant types of trends in land surface fluxes among six monsoon sectors (Fig. 8a). The first is increasing LHF and decreasing SHF (that is, (LHF+, SHF−)), which occurs in 41.6% land area in the global monsoon domains and is the dominant trends in monsoon sectors ASN, AUS, NAM, NAF. This is consistent with the increasing moisture gradient and decreasing temperature gradient between land surface and 2-m air in monsoon domains based on Era-interim reanalysis dataset^36^ (Fig. S2a,b). In contrast, the trend (LHF−, SHF+) is observed in only 17.8% land area and is not dominant in any monsoon sector. The second is the (LHF+, SHF+), which is observed in 24.5% of land area (including center and east of SAM, center SAF, and east of ASN) and dominant in SAM. Notably, these regions all locate within the rainforest. The third is the (LHF−, SHF−), which is observed in 11.7% of land area and dominates in the SAF.

**Figure 9:**
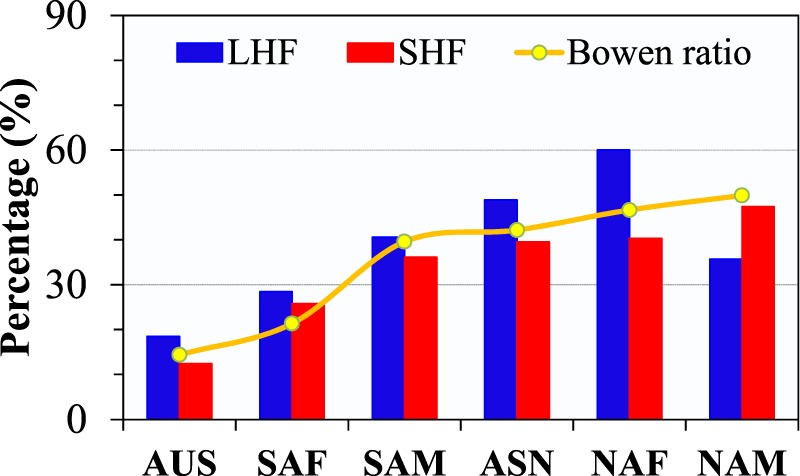
Percentages of land area that has statistically significant trend (*p* < 0.05) in LHF, SHF and Bowen ratio for six monsoon domains.

In addition, it is worth mentioning that not all of the trends in LHF, SHF and Bowen ratio are statistically significant. As presented in Fig. 9, trends in about 30% area in SAF are statistically significant (*p* < 0.05) and the percentage is even lower than 20% in AUS. For the other four monsoon sectors, the percentages of significant trends range from 36% to 60%. By comparison, fractions of significant trends in LHF are higher than that SHF and Bowen ratio in all monsoon sectors except the NAM.

**Figure 10:**
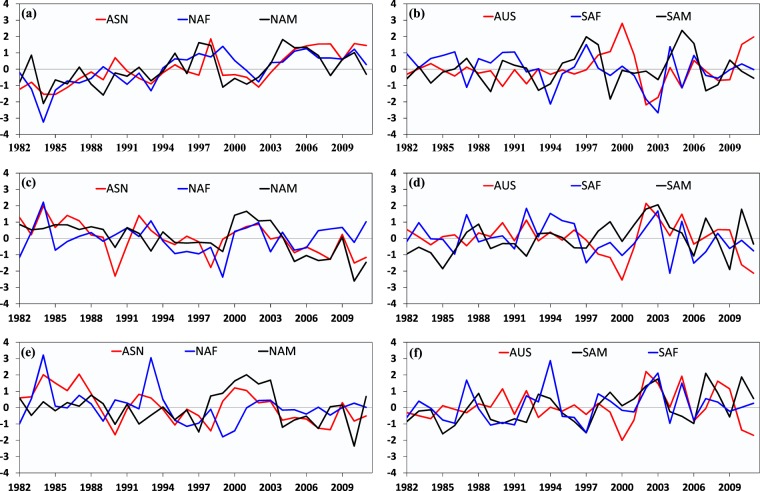
Variations in LHF (**a**,**b**), SHF (**c**,**d**), and Bowen ratio (**e**,**f**) for six monsoon sectors in Northern and Southern Hemisphere. For comparison, here the LHF, SHF and Bowen ratio are normalized by their maximums (that is, the maximums of time series) in each monsoon sector.

Moreover, the trends in monsoon domains are different between the NH and SH. The percentages of significant trends in LHF, SHF and Bowen ratio are all higher in NH monsoon domains (Fig. 9). As listed in Table 2, the trends for NH monsoon domains are at least one order of magnitude larger than that for SH monsoon regions, which is partly attributed to the opposite trend signs in SH monsoon sectors. We further investigate the regional average variations in LHF, SHF and Bowen ratio for six monsoon sectors. As presented in Fig. 10, no obvious trends are observed for monsoon sectors in SH, while significant increased trends in LHF and decreased trends in SHF and Bowen ratio are observed in NH. This may be related to the variability of monsoonal precipitation. The global monsoonal precipitation has significantly increased in the recent decades, especially in the NH, mainly due to the significant rising trend of NH summer monsoon^9–11^. This is also partly associated with the intensified and weakened land surface wind speed in NH and SH monsoon domains, respectively (Fig. S2c). More notably, in contrast to monsoon regions, drylands experience opposite trends for both LHF and SHF, with a decreased trend in LHF and an increased trend in SHF (Fig. 6). This indicates LHF is becoming richer in monsoon regions but poorer in drylands, which is in line with the observed wet-get-wetter and dry-gets-drier precipitation trend patterns over the past 30 years^10^.

**Table 2:** Average trends in LHF, SHF and Bowen ratio for Northern Hemisphere (NH) and Southern Hemisphere (SH) during 1982 and 2011.

	NH	SH
LHF (MJ m^−2^ yr^−1^ yr^−1^)	2.5E + 00	1.2E − 02
SHF (MJ m^−2^ yr^−1^ yr^−1^)	−6.5E − 01	6.1E − 02
Bowen ratio (yr^−1^)	−2.2E − 03	5.6E − 05

## The Influences of Monsoon Strength and Precipitation on Land Surface Energy Partitioning

In monsoon domains, monsoon dominates the regional climate. Monsoonal precipitation provides the majority of fresh water for ecosystems and exerts significant influence on the land-atmosphere energy exchanges. With the global warming, the global monsoon and monsoonal precipitation have significantly increased in the recent decades (Fig. 3). As discussed above, the land surface fluxes in monsoon domains shows three dominant trend patterns, including the (LHF+, SHF−), (LHF+, SHF+) and (LHF−, SHF−). These variations in land surface fluxes may be connected to the intensifying monsoon and monsoonal precipitation. Evidently land surface conditions such as soil moisture are also critical for understanding the variation of land surface heat fluxes, but these thermal-hydrologic properties of land surface are significantly regulated by climate and circulations, such as monsoon and precipitation (Figs. S3,S4). Here we emphasize the responses of heat fluxes to monsoon and precipitation in monsoon domains. In this section, the singular value decomposition (SVD) analyses are applied to decompose the covariability between precipitation and LHF and SHF to assess the influences of monsoon and monsoonal precipitation on land surface energy partitioning. We focus on the spatial patterns of first modes for monsoon strength, precipitation, LHF and SHF, which are acquired by correlating them with the corresponding time series.

### Coherent variations of precipitation and LHF, SHF and Bowen ratio

**Figure 11:**
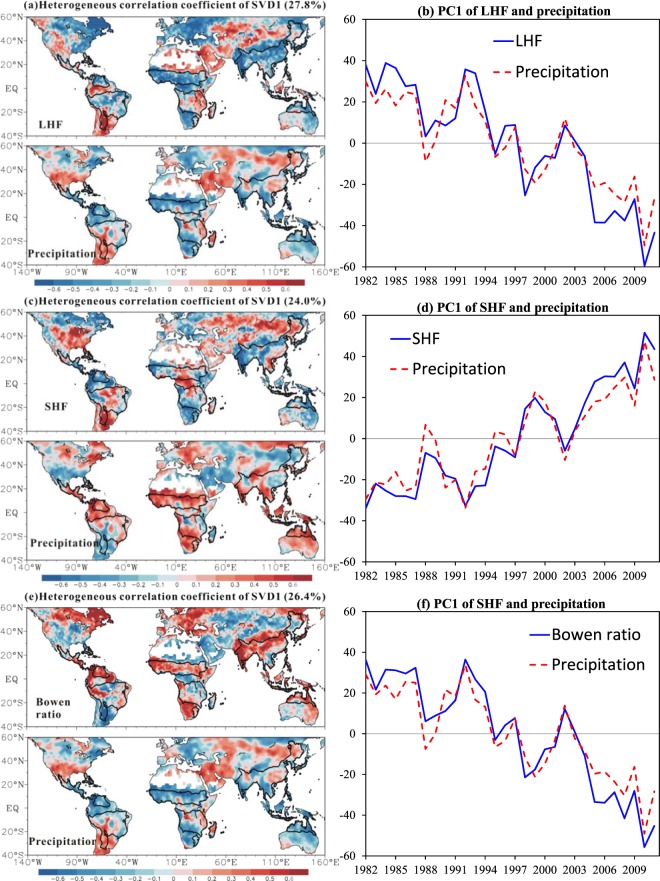
The primary modes of SVD analysis between mean annual LHF, SHF, and Bowen ratio and precipitation for the period of 1982–2011. (**a**,**c**,**e**) the heterogeneous correlation coefficient patterns; (**b**,**d**,**f**) the corresponding time series. The explained covariance is given in the parentheses above (**a**,**c**,**e**).

**Figure 12:**
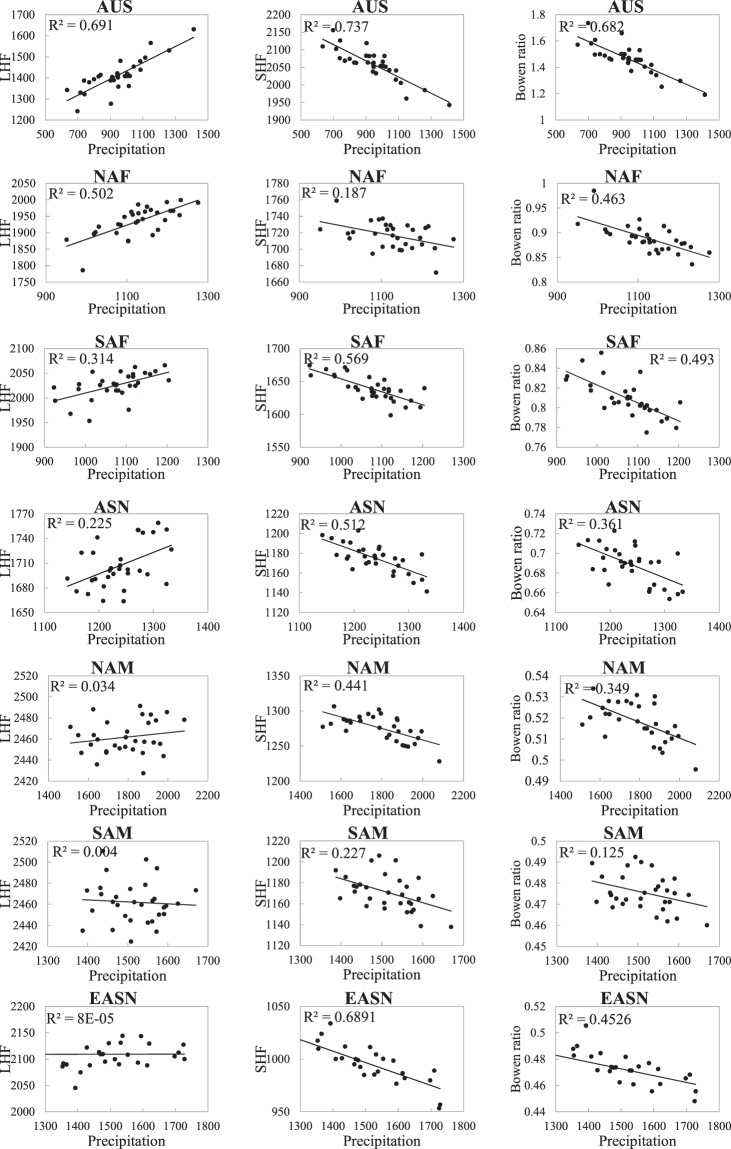
Relations between annual precipitation (in mm yr^−1^) and LHF (left panel, in MJ m^−2^ yr^−1^), SHF (middle panel, in MJ m^−2^ yr^−1^), and Bowen ratio (right panel) among monsoon domains for the period of 1982–2011.

**Figure 13:**
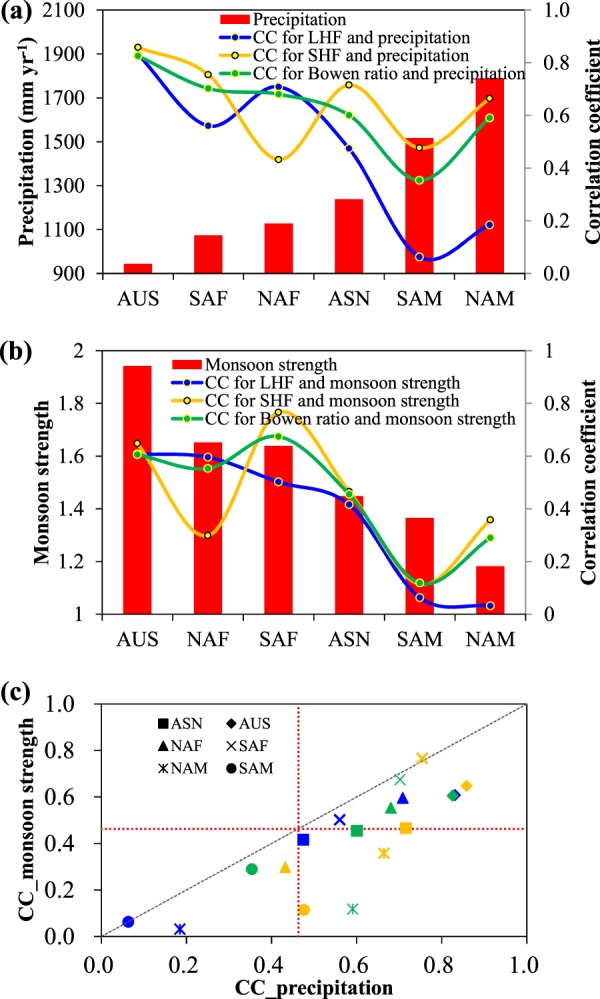
The Correlation coefficients (CCs) of LHF (blue), SHF (yellow) and Bowen ratio (green) with precipitation and monsoon strength for six monsoon sectors. (**a**,**b**) The precipitation and monsoon strength are shown by red column. (**c**) The x-axis and y-axis are the CCs related to precipitation and monsoon strength, respectively. The CCs for each sector are marked with different symbols. The grey dashed line is the 1:1 line. The red dashed lines denote the CC threshold that is statistically significant (*p* < 0.01) based on student’s t-test.

The first SVD mode between annual precipitation and LHF explains 27.8% of total covariance, which is much higher than that of the second mode (10.8%) and third mode (8.3%). As displayed in Fig. 11a, the pattern of LHF shows high spatial consistency with that of annual precipitation. Similar spatial coherence between precipitation and LHF trends are also reported by previous study^37^. The increase (decrease) in LHF is related to the increase (decrease) in precipitation. This is reasonable because precipitation can increase the humidity difference between the land surface and the near-surface atmosphere. Moreover, the spatial pattern of the first SVD mode of LHF in monsoon domains is highly matched by the pattern of LHF trends. This on one hand indicates that increased trends shown in Fig. 6a capture the primary variability of LHF; on the other hand it suggests that precipitation is one of main mechanisms contributing to the rising trends in LHF in monsoon sector ASN, NAF, NAM and AUS and the decreased trend for LHF in SAF. However, the eastern ASN (EASN) shows an increase in LHF with decreased precipitation, which agrees with findings of Qian *et al*.^38^. The possible reason is that precipitation has weak impact on evapotranspiration variability in some wet climate regimes where the evapotranspiration is radiation-limited. In fact EASN is not the only exception. Figure 12 presents the relations between annual LHF and precipitation on regional scale. The correlation coefficients between LHF and precipitation are 0.831, 0.709, 0.560, 0.474, 0.184, 0.063 and 0.009 for AUS, NAF, SAF, ASN, NAM, SAM and EASN respectively. One can see that the linear correlation between LHF and precipitation is much higher and statistically significant (*p* < 0.01) in AUS, NAF, SAF, and ASN, where the mean regional precipitation is below 1300 mm yr^−1^. No significant correlation, however, is observed in EASN, SAM and NAM, where the mean regional precipitation is higher than 1400 mm yr^−1^. The weak precipitation-LHF connection is also partly attributed to run-off, especially in SAM and NAM where part of precipitation runs into the wide-stretched Amazon River and the run-off increases remarkably during 2000s (Fig. S5). Consequently, it seems LHF does not always linearly respond to precipitation, but exhibit a threshold feature (Fig. 13a), which resembles the soil moisture-evapotranspiration coupling^7^.

The first SVD mode between annual precipitation and SHF accounts for 24.0% of total covariance, much higher than the second (10.6%) and third (7.6%) modes. Contrary to LHF, the spatial pattern of the first mode of SHF is oppositely matched by that of precipitation (Fig. 11c). The increase (decrease) in SHF is closely related to the decrease (increase) in precipitation in all monsoon sectors. Similar to LHF, the spatial pattern of the first mode of SHF is also consistent with the trend patterns shown in Fig. 6b, which further confirms the role of precipitation played in the trends of SHF. Moreover, SHF is more sensitive than LHF to the variation in precipitation (Fig. 12). The correlation coefficients between SHF and precipitation are 0.858, 0.432, 0.754, 0.716, 0.664, 0.476, and 0.830 for AUS, NAF, SAF, ASN, NAM, SAM, and EASN respectively, which are statistically significant (*p* < 0.01) and are higher than the correlation coefficients between LHF and precipitation except NAF (Fig. 13c). However, the SHF−precipitation correlation is not sensitive to the precipitation amount itself (Fig. 13a). SHF shows strong linear response to precipitation even in wet climate regimes such as SAM, NAM and EASN, which is different from LHF.

Similar to SHF, the spatial pattern of the first mode of Bowen ratio is also oppositely matched by that of precipitation (Fig. 11f), which accounts for 26.4% of total covariance. The increase (decrease) in Bowen ratio is closely related to the decrease (increase) in precipitation. Moreover, Bowen ratio shows strong negative correlation with precipitation in all monsoon domains (Fig. 12), with the correlation is significant at 99% level except SAM (Fig. 13c). In addition, the Bowen ratio-precipitation correlation is sensitive to the precipitation amount itself. But there seems no threshold feature is observed that appears in the LHF-precipitation correlation (Fig. 13a).

Therefore, the trend pattern (LHF+, SHF−) dominant in ASN, NAF and AUS and the trend (LHF−, SHF−) dominant in SAF are sensitive to the variation in precipitation. However, the trend patterns (LHF+, SHF+) that dominates in the SAM and (LHF+, SHF−) that dominates in NAM only partially responds to precipitation. The SAM and NAM have excessive precipitation and are covered by a wide stretch tropical rainforest (that is, the Amazon rainforest), which makes their LHF energy-limited and not sensitive to the variability in precipitation. In addition, the excessive precipitation may be largely turn into run-off and thus will not participate in evaporation over land. Moreover, as shown in Fig. 8b, the (LHF, SHF) pattern based on the heterogeneous correlation coefficient patterns of SVD first modes highly matches the (LHF, SHF) trend pattern showed in Fig. 8a. The spatial consistency indicates precipitation indeed exerts significant impacts on the land surface energy partitioning in monsoon domains.

The first principal components of the first modes of SVD analysis between precipitation and LHF, SHF and Bowen ratio are also presented (Fig. 11b,d,f). Dramatic phase shifts of the first principal components are observed in the late-1990s. Notably, it concurs with the strong ENSO event during 1998–1999 and the shift in ENSO^39^. Both LHF and precipitation shift from negative to positive phases in monsoon domains. In contrast, SHF was in positive phase before late-1990s and in negative phase afterwards. Notably, phase shifts in LHF and SHF are also observed in non-monsoon domains. But the shifts for the drylands are in opposite directions to those in monsoon domains (Figs. 11a,c). Consequently, the phase shifts of land surface energy partitioning occur globally in response to variation of precipitation under the global warming, but they seem contrary in monsoon domains and drylands. These shifts might be controlled by natural variability. The Atlantic multidecadal oscillation has also experienced a remarkable phase shift from previous negative phases to post positive phases around the mid-1990s^40,41^.

### The relations between monsoon and LHF, SHF and Bowen ratio

**Figure 14:**
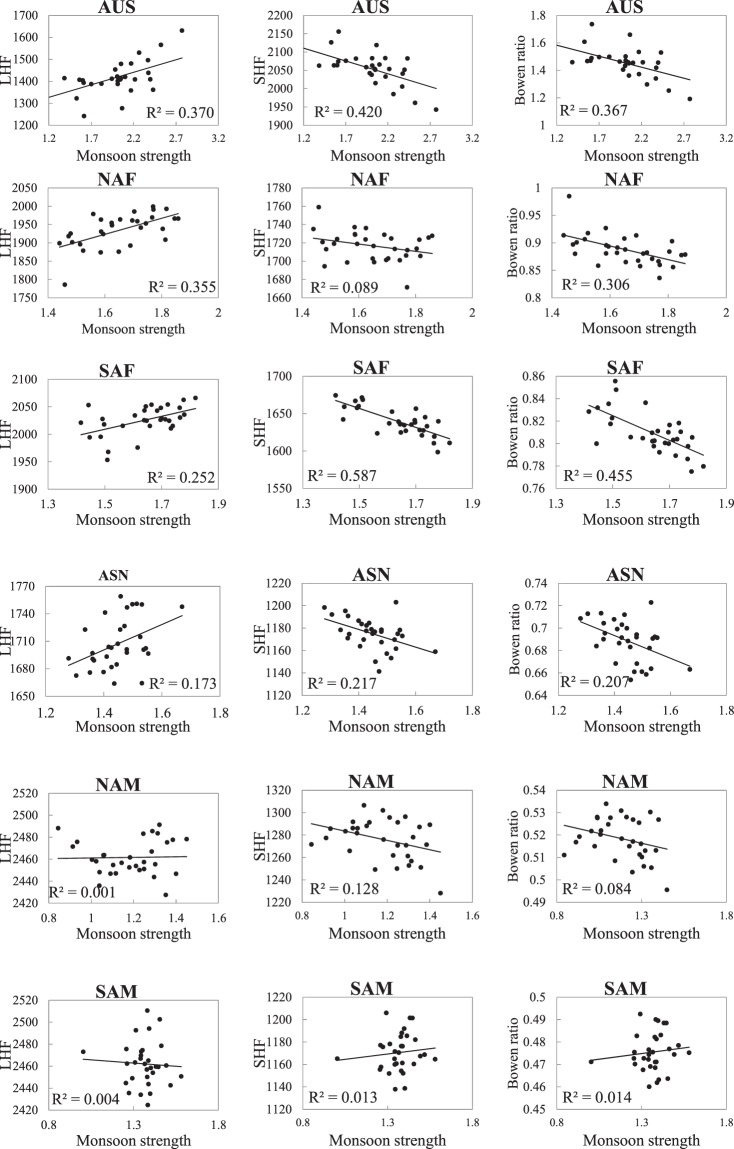
Relations between monsoon strength and LHF (left panel, in MJ m^−2^ yr^−1^), SHF (middle panel, in MJ m^−2^ yr^−1^), and Bowen ratio (right panel) among monsoon domains for the period of 1982–2011.

In the monsoon domains, monsoon strength has significant control on precipitation and thus influences on the land surface fluxes (Fig. 3). SVD analysis is also performed on monsoon strength and LHF, SHF and Bowen ratio. The results resemble those of precipitation and thus no figures are presents. The first SVD modes (figures not shown) between monsoon strength and LHF, SHF and Bowen ratio explain 25.2%, 22.2% and 24.0% of total covariance, respectively. The spatial patterns of the first modes of LHF, SHF and Bowen ratio closely match that of monsoon strength. On regional scale, similar to precipitation, monsoon strength also significantly (*p* < 0.01) correlates with LHF and SHF (Figs. 13c and 14). The stronger monsoon often results in larger LHF and smaller SHF. However, the LHF-monsoon strength correlation shows high sensitivity to monsoon strength itself (Fig. 13b). No obvious relation between LHF and monsoon is observed in weak monsoon domains NAM and SAM where weak response of LHF to precipitation is also observed. But there exist some differences in the responses of land surface fluxes to monsoon strength and precipitation. For example, monsoon seems to have weak constraints on SHF in SAM and NAM where SHF significantly correlates with precipitation. Moreover, the responses of LHF, SHF and Bowen ratio to monsoon strength are less robust than to precipitation (Fig. 13c), which is primarily attributed to the fact that precipitation exerts more direct influences on land surface energy partitioning.

## Summary

The land surface LHF and SHF are key energy sources that drive atmosphere circulation, and thus are closely linked with weather and climate changes. In this paper, we investigate the long-term mean, trends and leading modes of land surface LHF, SHF and Bowen ratio in global land monsoon domains. The responses of LHF, SHF and Bowen ratio to monsoon and precipitation are also discussed.

As expected, LHF dominated in monsoon domains and the mean LHF is about one quarter larger than SHF. During the past three decades, LHF and SHF show three dominant trend patterns in global terrestrial monsoon domains. The first is (LHF+, SHF−) dominated in ASN, AUS, NAF, NAM monsoon sectors. Moreover, the increasing rate of LHF was higher than the decreasing rate of SHF, which resulted in a decreased trend in Bowen ratio. This implies the energy needed by increased LHF is only partially due to the decreased SHF and the remaining is associated with an increase in land surface net radiation. Similar relations among LHF, SHF and net radiation are discussed over urban land surfaces^42^. The trend patterns (LHF+, SHF+) and (LHF−, SHF−) are dominated in SAM and SAF, respectively. In contrast, (LHF−, SHF+) is dominated only in drylands. These trends result in remarkable phase shifts in LHF and SHF in both monsoon domains and drylands during late-1990s. LHF shifts from negative to positive phase and SHF from the positive to negative in the monsoon domains. But the shifts are in reversed direction in drylands. The distinctions between monsoon domains and drylands suggest that climate is generally becoming wetter in monsoon regions but drier in drylands with global warming during the past three decades.

The variability and regional difference in LHF, SHF and Bowen ratio are found to be closely linked to precipitation and monsoon strength. On regional scale, precipitation significantly positively correlates with LHF in AUS, NAF, SAF and ASN where the mean annual precipitation is below 1300 mm yr^−1^, but weakly correlates with LHF in NAM, SAM and EASN where the mean annual precipitation is over 1400 mm yr^−1^. It seems the response of LHF to precipitation is weak in more humid monsoon climates but shows strong linearity in less humid monsoon climates. Significant negative correlations between precipitation and SHF and Bowen ratio are observed in all monsoon sectors. Moreover, SVD analyses show that precipitation explains 27.8%, 24% and 26.4% total covariance of LHF, SHF and Bowen ratio, respectively, in the primary modes. The spatial patterns of the first modes of LHF, SHF and Bowen ratio are closely matched by that of precipitation, which indicates the strong regulation of precipitation on land surface energy partitioning on pixel scale. Precipitation on one hand favors more land surface available energy being converted into LHF, on the other it enhances the LHF by increasing the land surface net radiation. The intensifying LHF and precipitation means acceleration of hydrological cycle in global land monsoon domains, which is consistent with previous studies and may have substantial impact on the availability and distribution of water as well as the ecosystems upon which we depend^43,44^. Additionally, since precipitation and monsoon strength are closely related, the responses of LHF, SHF and Bowen ratio to monsoon strength resemble that to precipitation but less robust.

Finally, the work is based on the upscaled product of FLUXNET observations, which might introduce some uncertainties in regions where observations are rare or even unavailable. For instance, FLUXNET has very few observations from India, and the observation sites are sparse in Africa. The conclusions on trends of land surface heat fluxes in these regions should be taken with caution.

## Supplementary information

Supplementary figures.
