# Supplementary material for: The trends in land surface heat fluxes over global monsoon domains and their responses to monsoon and precipitation
Source: Sci Rep. 2020 Apr 1;10:5762. doi: 10.1038/s41598-020-62467-0 (PMC7113305; doi:10.1038/s41598-020-62467-0)
Supplement: Supplementary file 1 — Supplementary figures. [file 41598_2020_62467_MOESM1_ESM.pdf]

The trends in land surface heat fluxes over global monsoon domains and their responses to monsoon and precipitation

Jian Zeng,[a, b] Qiang Zhang,[a, b, c*]

[a] *College of Atmospheric Sciences, Lanzhou University, Lanzhou, China*

[b] *Key Laboratory of Arid Climatic Change and Disaster Reduction in Gansu Province; Key Open Laboratory of Arid Climatic Change and Disaster Reduction in CMA; Institute of Arid Meteorology, CMA, Lanzhou, China*

[c] *Gansu Provincial Meteorological Bureau, Lanzhou, China*

***Corresponding author: Qiang Zhang, zhangqiang@cma.gov.cn; Qzhang@ns.lzb.ac.cn**

Postal address:

College of Atmospheric Sciences

Lanzhou University

222 South Tianshui Road

Lanzhou 730000, Gansu Province

China

Email: zengj17@lzu.edu.cn

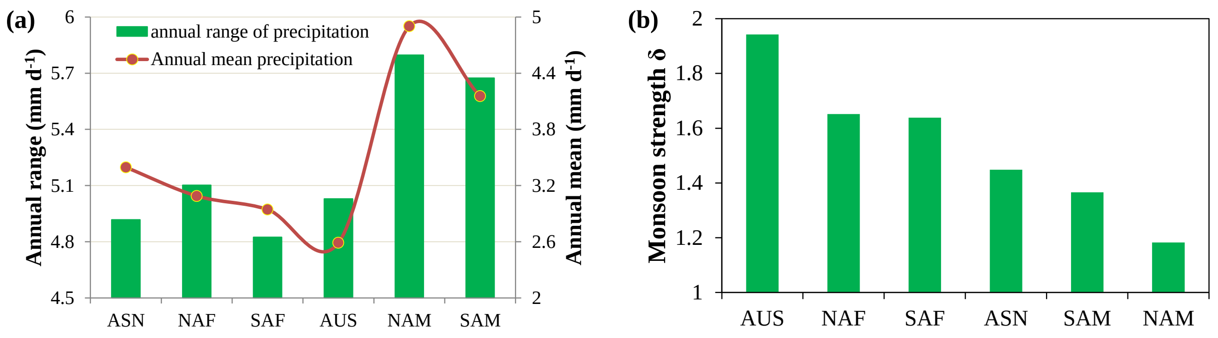

Fig. S1 The annual range of precipitation and annual mean precipitation (a), and monsoon strength δ (b) in six monsoon domains.

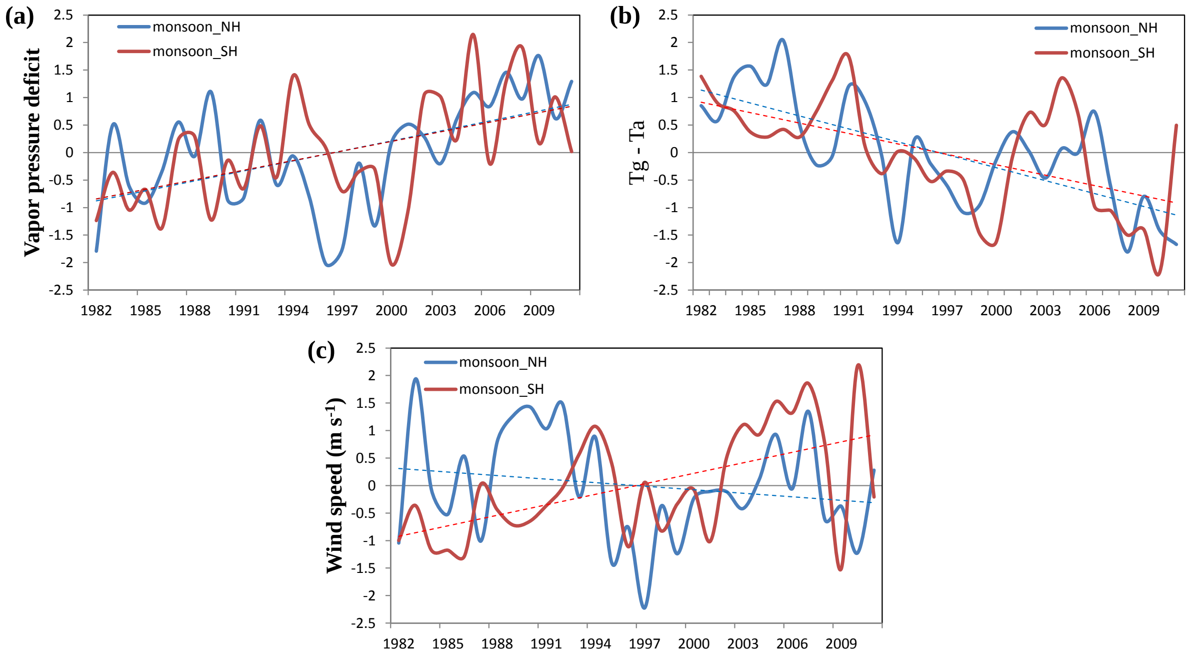

Fig.S2. Variations of normalized 2m vapor pressure deficit (a), temperature difference between 2m air and land surface (b), and 10m wind speed (c) monsoon domains in northern hemisphere (NH) and southern hemisphere (SH) during 1982-2011. The data used are derived from Era-interim reanalysis dataset.

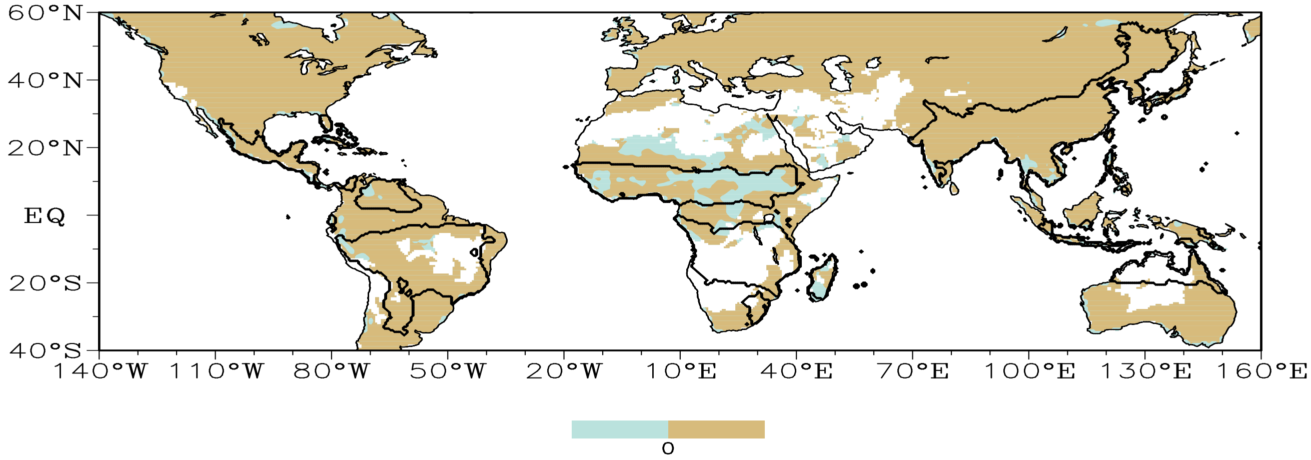

Fig.S3. Regression of annual precipitation and mean soil moisture derived from Era-interim reanalysis dataset.

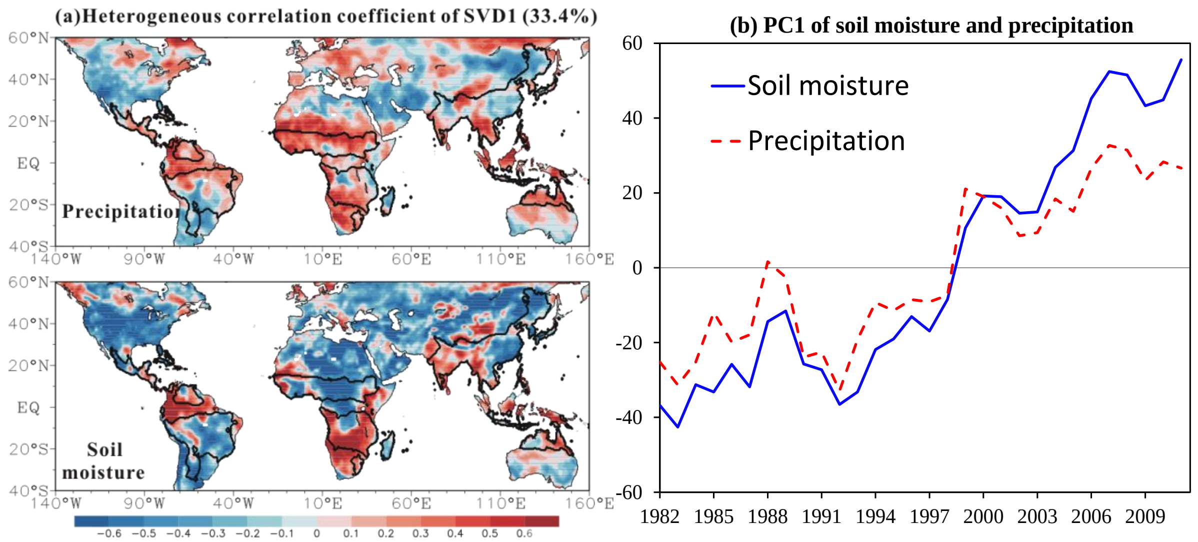

Fig.S4 The primary modes of SVD analysis between mean soil moisture and annual precipitation for the period of 1982–2011. (a) the heterogeneous correlation coefficient patterns; (b) the corresponding time series. The explained covariance is given in the parentheses above (a).

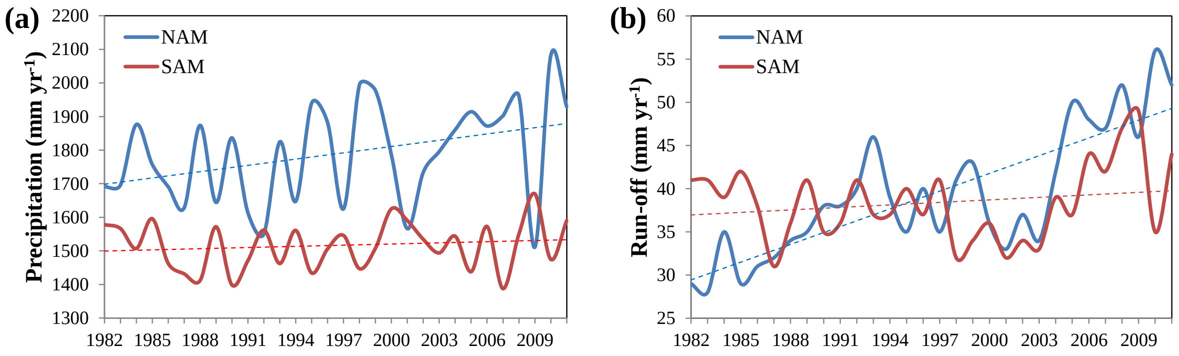

Fig.S5 Variations in precipitation (a) and run-off (b) in NAM and SAM.
